# Impact of polycyclic aromatic hydrocarbon exposure on cognitive function and neurodegeneration in humans: A systematic review and meta-analysis

**DOI:** 10.3389/fneur.2022.1052333

**Published:** 2023-01-10

**Authors:** Jessica Humphreys, Maria del C. Valdés Hernández

**Affiliations:** ^1^College of Medicine and Veterinary Medicine, University of Edinburgh, Edinburgh, United Kingdom; ^2^Centre for Clinical Brain Sciences, University of Edinburgh, Edinburgh, United Kingdom

**Keywords:** polycyclic aromatic hydrocarbon (PAH), cognition, neurological, neurodegeneration, neurobehavioral, meta-analysis, systematic literature search

## Abstract

**Introduction:**

This article documents an emerging body of evidence concerning the neurological effect of polycyclic aromatic hydrocarbon (PAH) exposure with regard to cognitive function and increased risk of neurodegeneration.

**Methods:**

Two electronic databases, PubMed and Web of Science, were systematically searched.

**Results:**

The 37/428 studies selected included outcomes measuring cognitive function, neurobehavioral symptoms of impaired cognition, and pathologies associated with neurodegeneration from pre-natal (21/37 studies), childhood (14/37 studies), and adult (8/37 studies) PAH exposure. Sufficient evidence was found surrounding pre-natal exposure negatively impacting child intelligence, mental development, average overall development, verbal IQ, and memory; externalizing, internalizing, anxious, and depressed behaviors; and behavioral development and child attentiveness. Evidence concerning exposure during childhood and as an adult was scarce and highly heterogeneous; however, the presence of neurodegenerative biomarkers and increased concentrations of cryptic “self” antigens in serum and cerebrospinal fluid samples suggest a higher risk of neurodegenerative disease. Associations with lowered cognitive ability and impaired attentiveness were found in children and memory disturbances, specifically auditory memory, verbal learning, and general memory in adults.

**Discussion:**

Although evidence is not yet conclusive and further research is needed, the studies included supported the hypothesis that PAH exposure negatively impacts cognitive function and increases the risk of neurodegeneration in humans, and recommends considering the introduction of a variable “rural vs. urban” as covariate for adjusting analyses, where the neurological functions affected (as result of our review) are outcome variables.

## Introduction

Exposure to air pollution in the environment is now recognized globally by governments, leading research scientists, and civil society as one of the greatest public health hazards of the 21st century (1). Legislation such as “The UK National Air Quality Strategy” (2), and the European Commission's “Fourth Daughter Directive” (3) have introduced standards to monitor and limit levels of air pollutants posing the greatest risk to human health. Polycyclic aromatic hydrocarbons (PAHs) are a group of pollutants included in such legislation. PAHs are atmospheric organic compounds composed of two or more benzene rings arranged in a variety of different configurations. PAH compounds also include functional derivatives of the PAHs only containing carbon and hydrogen atoms (e.g., nitro-PAHs) and the heterocyclic analogs (e.g., aza-arenes) (4). Over 100 different PAHs were already identified by the beginning of the 21st century (4), and now the list exceeds 300, with an exact number still to be determined, as those studied are mainly selected based on the instrumentation available to each research group and reference standards (5). They are discharged from anthropogenic sources (Supplementary Figure 1), involving the incomplete combustion and pyrolysis of hydrocarbons, predominantly found in coal, oil, wood, and petrol. PAHs exist in the atmosphere in a gaseous state or are adsorbed to particulate matter. Over 80% of particulate-bound PAHs are associated with particulate matter of an aerodynamic diameter ≤ 2.5 μm (PM_2.5_) (6). However, a large number have been also identified in tobacco smoke (5). The study of PAHs and their impact on health has been compounded by their ubiquitousness and the numerous and widespread sources in which they can be found, some of which are also affected by other air pollutants. As PAHs are rather present as part of complex mixtures in air, water, soil, and food, their identification and characterization, for studying their effect on human health, is challenging (5).

Research surrounding PAH exposure and acute short-term health effects in humans has, thus, far focused on vulnerable individuals with pre-existing health conditions: thrombotic effects in individuals with pre-existing coronary heart disease and impaired lung function in asthma sufferers (7). Chronic long-term exposure has implicated PAH's reactive metabolites as having the ability to bind to proteins and DNA and exert carcinogenic effects (8). Such biochemical disruption and cellular damage have been most extensively researched in occupational studies, whereby high exposure has been associated with increased incidence of lung, bladder, skin, and gastrointestinal cancer (8–11). Additionally, decreased immune function, developing cataracts, and having kidney or liver damage, including jaundice, have also been associated with high exposure (5, 12). Whilst extensive research exists surrounding PAH's genotoxic and carcinogenic properties, an emerging body of evidence concerns PAH's neurotoxic effect through the induction of oxidative stress, inflammation (13), and vascular injury within the brain (14). Recently, research has emerged associating PAH exposure with impaired cognitive function and increased risk of neurodegeneration.

To the best of our knowledge, from the large body of literature on the influence of air pollution on human health, the implications of PAH exposure specifically, on cognitive function and neurodegeneration in humans, have not been systematically reviewed. Prior reviews have addressed the implications of PAH exposure on general health (15, 16) and its carcinogenic outcomes (17, 18). The reviews which have made cognitive function and neurodegeneration the outcome of interest include exposures to a vast mixture of air pollutants (19–22). Therefore, we aim to disentangle the unique neurotoxic effect of PAHs in specific age groups and cognitive-related functions to provide evidence for cognitive research and more vigilant monitoring and tighter restrictions on the main sources of emission, tailored to each age group, given the differential factors affecting the various stages of brain development. The Department for Environment, Food and Rural Affairs currently considers annual monitoring of concentrations of one PAH, benzo(a)pyrene (B(a)P), to be a sufficient representation of all atmospheric PAHs, and classifies the potential effect on human health of PAHs collectively, as six compounds, categorized as probably or possibly carcinogenic. No mention is made of the adverse neurological impact (2). A possible explanation is the consideration of concentration levels that constitute a risk for cancer, below which the effect of these pollutants can pass inadvertently. The UK national air quality objective for B(a)P is 0.25 ng m^−3^. However, emissions of B(a)P have been increasing since 2008 and have exceeded this limit in multiple locations at multiple time points (23). Atmospheric PAH concentrations are subject to seasonal variation and climate (24), as seen in pollution level charts that are used by studies to stratify exposure. While such stratification may add granularity to the data, it is often unrealistic given urban movement and the effect of different local government policies e.g., transportation. A more robust stratification would be to contrast urban and rural areas, where the pollution levels known to widely differ. Therefore, a further aim is to explore the difference in PAH concentration in rural vs. metropolitan areas and the influence this could have on cognitive function and neurodegeneration to inform further studies.

## Methods

### Eligibility criteria

This review was conducted in line with the PRISMA guidelines (25). Studies included were observational cohort studies of both male and female humans. Time of exposure was inclusive of the gestational period and stretched throughout life until death. Exposure quantification was limited to studies that measured the level of exposure to ambient PAHs or PM_2.5_ through environmental air sampling or spatiotemporal modeling. Measures of exposure also included concentration of PAH metabolites in urine and dosimetry of PAH-DNA adducts from DNA extracted from white blood cells. Outcomes included involved a formal assessment of cognitive function, neurobehavioral symptoms of impaired cognition, and pathologies associated with neurodegeneration. Reports were limited to published scientific articles written in the English language. No publication dates were imposed. Studies were excluded if they did not fulfill the inclusion criteria, were not in humans, or where PAH exposure was measured as a component of the diet, environmental tobacco smoke (ETS), or traffic related air pollution (TRAP). Exposure through diet and ETS is not an appropriate representation of a major source of atmospheric PAH that can be geographically differential (i.e., in terms of urban vs. rural areas) or influential in both short- and long-term exposure. Moreover, prior research has elucidated contaminating pollutants within TRAP composition detrimentally affecting cognitive function, and the effect of diet-related benzo[a]pyrene, dibenz[a,h]anthracene, and benzo[h]fluoanthracene in human health and cognition (e.g., learning and memory functions). The inclusion of such studies would confound results and prevent us from elucidating the specific impact of ambient PAH on cognition.

### Information sources

Studies were identified by searching electronic databases PubMed (1984–2021) and Web of Science (1979–2021). Given the environmental changes seen as the consequence of lockdown policies and movement restrictions mainly in the period April 2020 to December 2021, publications that reflected or analyzed the environmental effect of this “abnormal” period were excluded. “Polycyclic aromatic hydrocarbons” in addition to the following search terms: “brain,” “neurological,” “cognitive,” “cognition,” “neurodegenerative,” “neurodegeneration,” “neurodevelopment,” and “neurodevelopmental” were used to identify articles in both databases. Limitations applied to the search included only the fields “Title” and “Abstract” being searched. In Web of Science, the document type “Articles” was applied. In PubMed, an additional limitation of species, “Humans,” was applied. Eligibility assessment was performed independently in an unbiased standardized manner by one reviewer. Ambiguity concerning the inclusion or exclusion of a study was resolved by a second reviewer being consulted and a consensus taken. Initial screening was performed by reviewing the title and abstract, after which, the full manuscript was reviewed.

### Data collection process

A data extraction sheet was developed and pilot tested on five randomly selected included studies, before being refined accordingly. One review author extracted data from the included studies, a second was consulted where ambiguity arose surrounding the appropriate data to extract. One author was contacted through email to provide numerical data that had only been presented graphically. Information extracted from studies comprised sample size, sample characteristics, ratio between sexes, mean age, age range, comorbidities, air pollution component, time of exposure, air pollution data acquisition method, and outcome measure.

### Risk of bias in individual studies

The risk of bias was assessed in line with the QUADAS^1^ guidelines (University of Bristol, 2003). To ascertain the risk of bias within each study included, one reviewer working independently extracted the following information: participant inclusion/exclusion criteria explained, participant withdrawals from the study explained, use of/comparison with a control/low exposure population, confounding variables identified, appropriate method/analysis to adjust for confounding variables, outcome assessors aware of exposure status of study participant, intermediate or unexpected results explained/reported, and whether or not the methods of the study were reproducible.

### Methods of analysis

Studies included were divided into four subgroups depending on the time at which the exposure was measured: pre-natal, childhood, adult, and finally childhood + adult. The category childhood + adult included studies of young individuals from a wide age range, some exposed during childhood, and others where exposure extended through to adulthood, not analyzed separately. Subsequently, studies were further divided into categories depending on the outcome measured: cognitive abilities, neurobehavioral development, or neurodegeneration. This subgroup division was conducted to adjust for heterogeneity between studies. The meta-analysis was performed by extracting odds ratios and 95% confidence intervals (CI) for the effect sizes of reported outcomes or calculating them from the parameters and data given in the original publications using the Practical Meta-Analysis Effect Size Calculator by David B. Wilson, from (https://www.campbellcollaboration.org/escalc/html/EffectSizeCalculator-OR-main.php). Results were double-checked using the following online resources: (https://www.gigacalculator.com/calculators/odds-ratio-calculator.php) and effect size converter (https://www.escal.site/). Forest plots were used to visualize differences in effect sizes between studies within the same subgroup.

## Results

### Study selection

**Figure 1:**
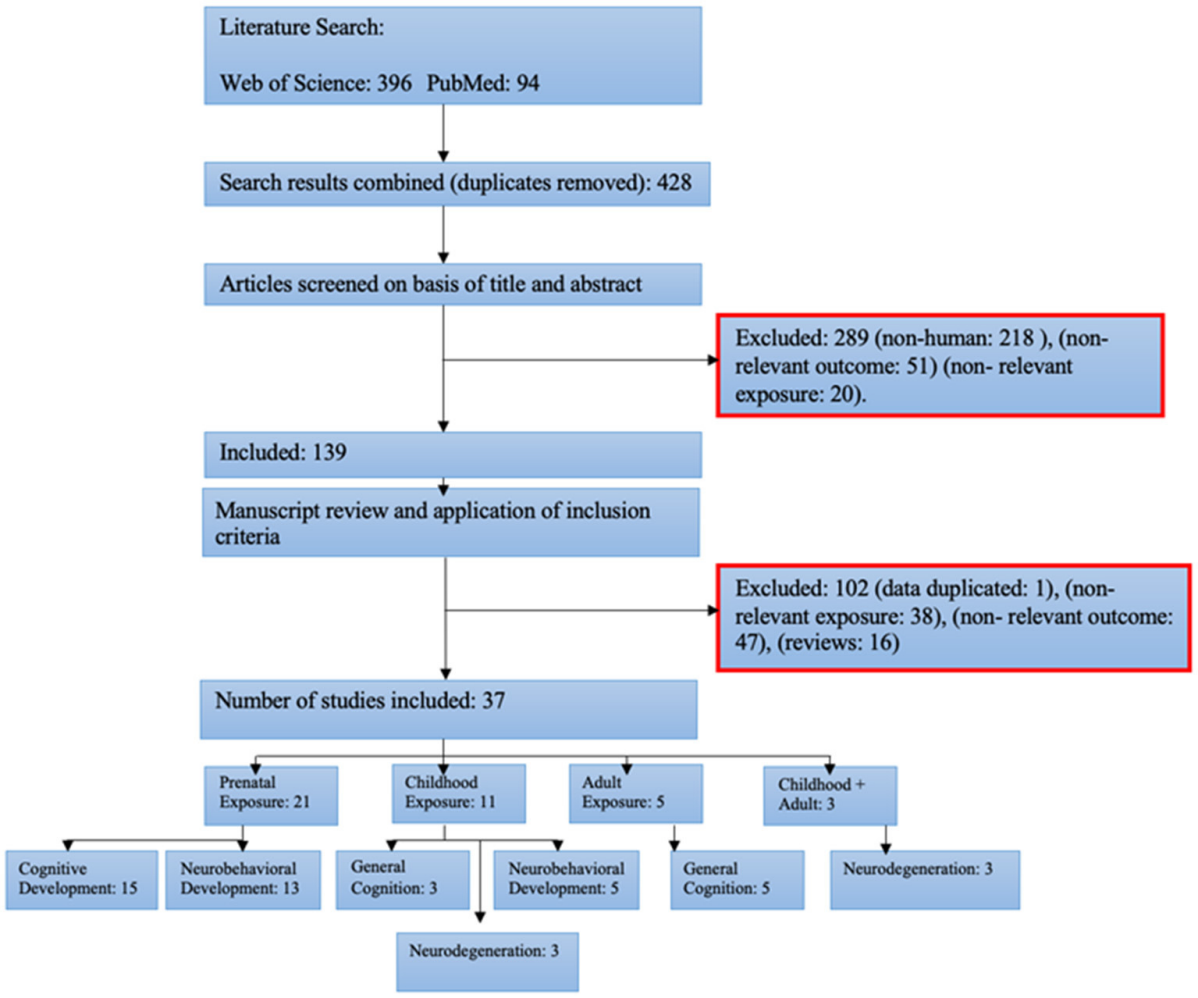
Flow chart of the search, study inclusion, and subgroup division.

The search of Web of Science and PubMed provided a total of 490 citations. After adjusting for duplicates, 428 remained. Subsequent screening of the title and abstract resulted in a further 289 being discarded. Of the remaining 139, a further 102 were excluded upon further examination of the full manuscript and application of inclusion criteria. One study (26) reported data from a previous study (27). Sixteen were reviews and did not include any primary data, 47 reported outcomes not relevant to cognitive function or neurodegeneration, and in 38 the recorded exposure to PAH was not in keeping with the specified criteria, resulting in a total of 37 studies included in this review. Subgroup division resulted in 21 pre-natal exposure studies, 15 concerning cognitive development outcomes, and 13 on neurobehavioral development, seven included measures of both. From 11 childhood exposure studies, three were on general cognition, five on neurobehavioral development, and three on neurodegeneration. There were also five adult exposure studies all with outcomes of general cognition, and three childhood + adult studies all with measures of neurodegeneration. One study included measured outcomes for both pre-natal and childhood exposure (28) and two studies involved two different study cohorts: one exposed only during childhood and the other included a mix of childhood and adult-exposed subjects (29, 30). Figure 1 depicts the flow chart for study inclusion and subgroup division. The full dataset can be found at the following link: https://doi.org/10.7488/ds/3031.

### Study characteristics

**Figure 2:**
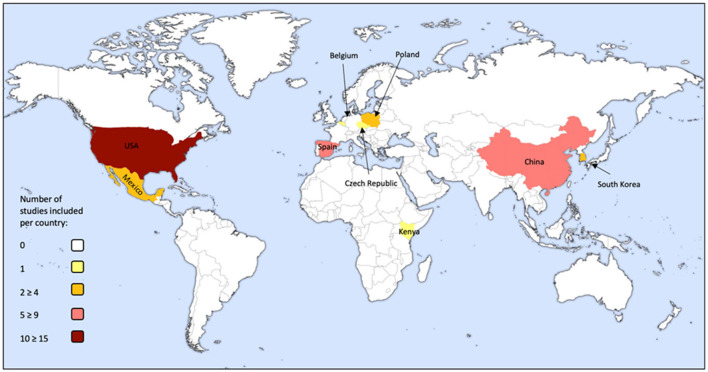
Global distribution of the population cohorts in each of the 37 studies included in this review (figure made using: Biorender.com).

**Figure 3:**
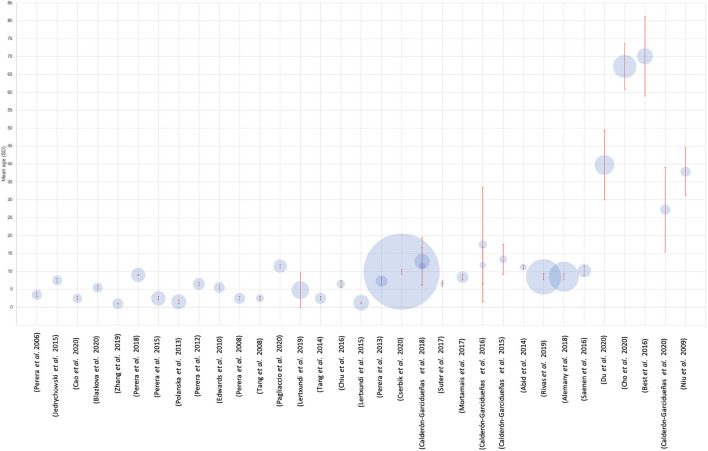
Characteristics of the study population involved in each study. Circle size is representative of the sample size. Red bars indicate mean age and SD. Four studies were omitted from this analysis due to insufficient data (28, 34, 43, 60).

The 37 studies involved populations from nine countries (Figure 2). Study population characteristics including sample size and mean age (±SD) are displayed in Figure 3.

Eleven studies included cohorts from the USA. Six of them selected participants from the Columbia Center for Children's Environmental Health cohort; however, each study selected different subgroups of the population and measured different outcomes (31–36). Two studies selected a subgroup of participants from the National Health and Nutrition Examination Survey 2001–2002 (NHANES) (37), one of which included additional participants from the NHANES 2003–2004 cohort (38). The remaining three studies involved cohorts from the Childhood Autism Risks from Genetics and the Environment Study (28), the Adolescent Brain Cognitive Development Study (39), and the Asthma Coalition on Community, Environment and Social Stress project (40). Eight studies reported results from populations in China. One study involved a Taiyuan population (41) in addition to two selecting different subgroups from the Taiyuan Mother and Child Cohort Study (42, 43). Three involved populations were from Tongliang (27, 44, 45), and the remaining two were from Shanxi province (46) and Qingdao City (47).

Five studies involved populations from Spain. Two involved a subgroup from the Infancia y Medio Ambiente Project (48, 49) and three studies from the Brain Development and Air Pollution Ultrafine Particles in School Children project (50–52).

Four studies reported on populations in Poland, three including participants from the Krakow Study (53–55) and one from the Polish Mother and Child Cohort Study (56). Four studies reported on populations in Mexico. All involved Mexico City residents (57), where two refer to six Mexican cities (29, 58), and another included details of three small Mexican cities (30). Two studies involved a Korean population (59, 60). Further individual studies included populations from the Czech Republic (61), Kenya (62), and Belgium (63).

### Exposure assessment

**Figure 4:**
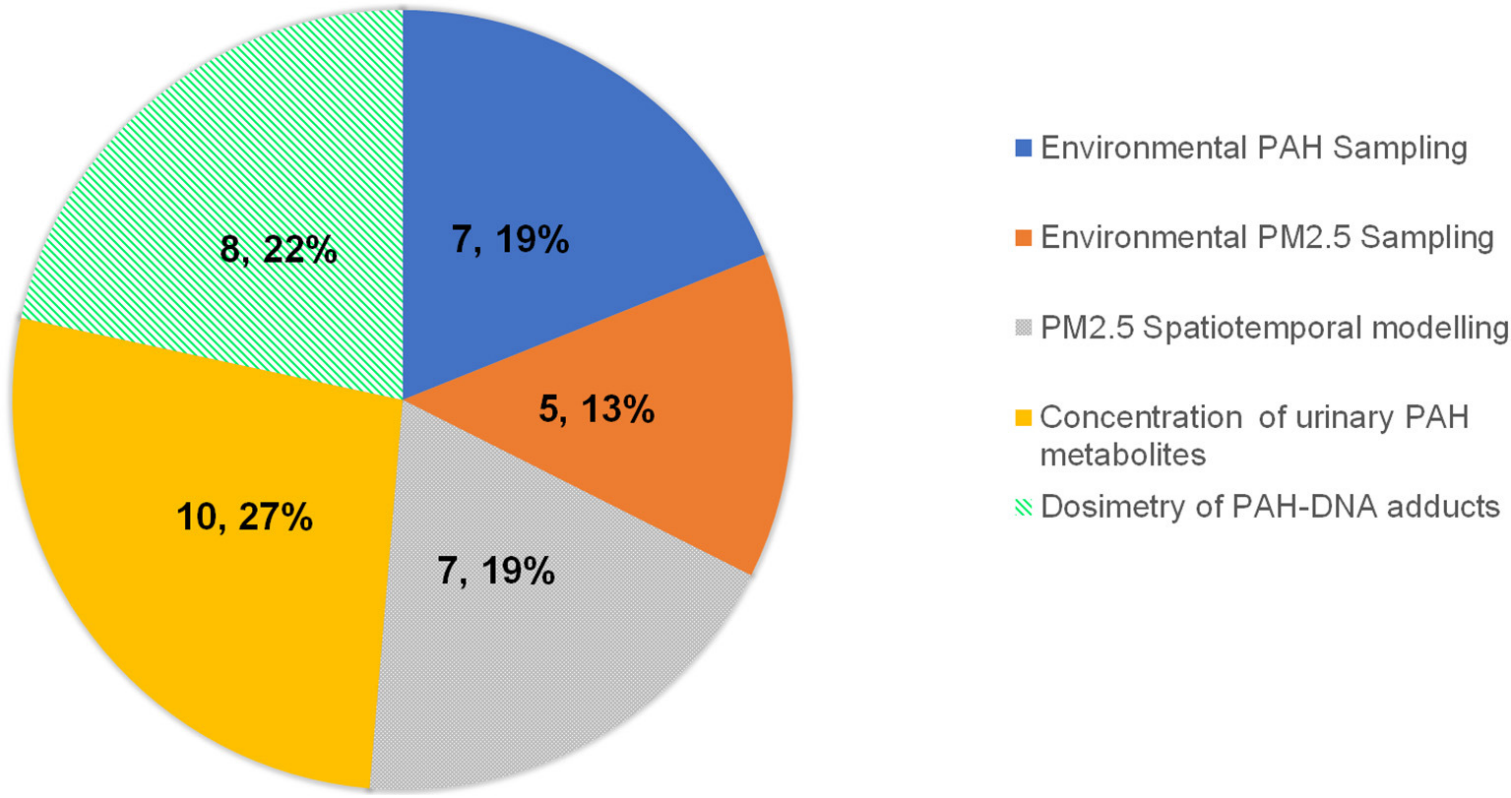
Pie chart representing the proportion of included studies measuring exposure to PAH as a measure of environmental PAH sampling, environmental PM_2.5_ sampling, PM_2.5_ spatiotemporal modeling, concentration of urinary PAH metabolites, and dosimetry of PAH-DNA adducts.

Of the 37 studies included, seven measured exposure through environmental PAH sampling, five by environmental PM_2.5_ sampling, seven by PM_2.5_ spatiotemporal modeling, 10 by concentrations of PAH metabolites in urine, and eight using dosimetry to measure PAH-DNA adducts (Figure 4).

### Outcome assessments

**Figure 5:**
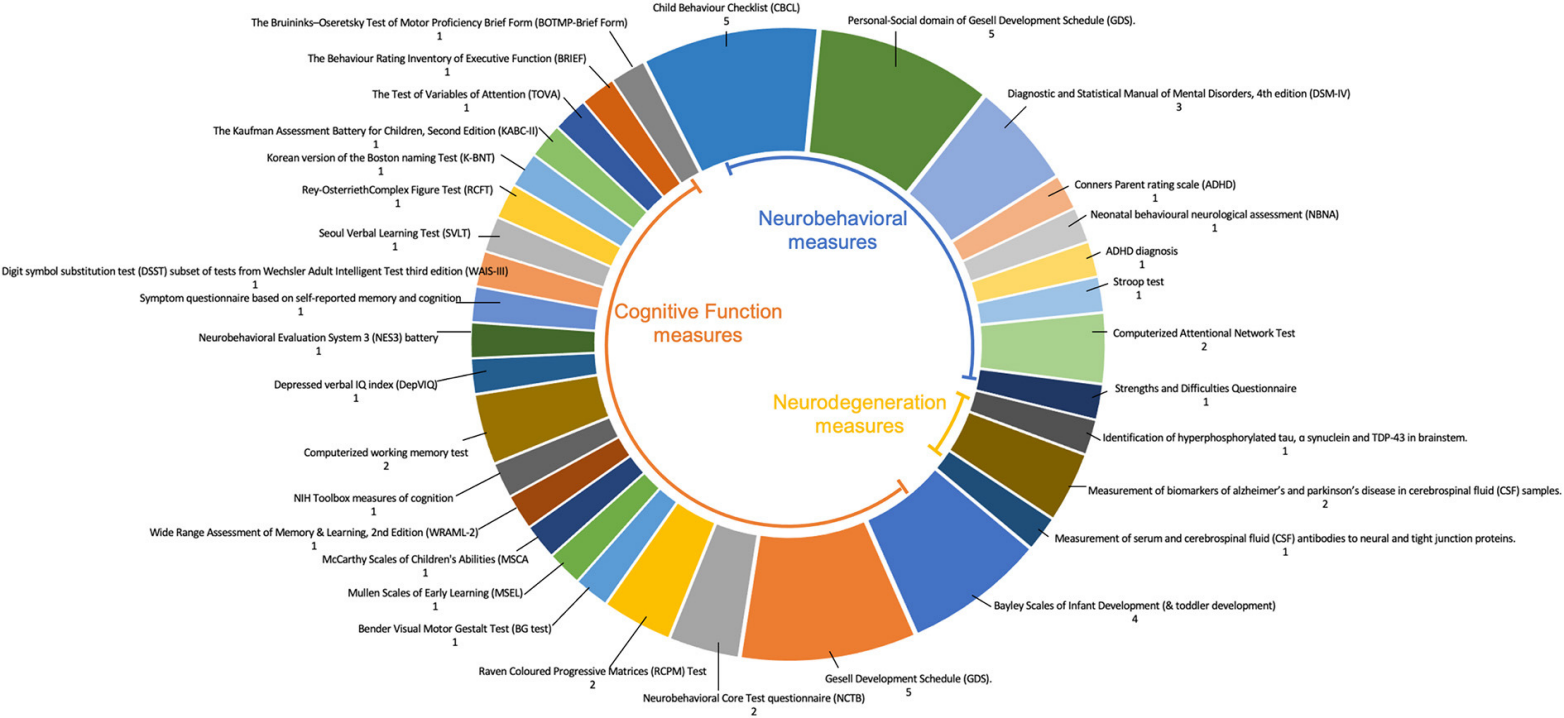
Pie chart representing the number of studies using different tests to measure outcomes. Measures include cognitive function, neurobehavioral symptoms of impaired cognition, and pathologies associated with neurodegeneration.

Outcomes included 21 different tests measuring cognitive function, nine different tests measuring neurobehavioral symptoms of impaired cognition, and three different measures of pathologies associated with neurodegeneration (Figure 5).

### Pre-natal exposure

#### Association between pre-natal PAH exposure and cognitive abilities in childhood

**Figure 6:**
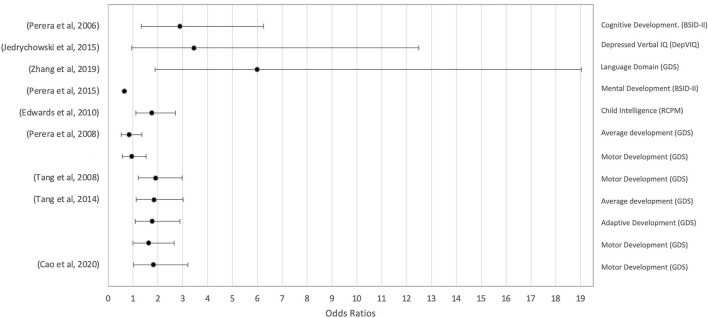
Forest plot of the calculated odds ratios (OR) and 95% confidence intervals (95% CI) for the association between pre-natal exposure to PAH and measures of cognitive development. Bayley Scales of Infant Development—revised (BSID-II), Depressed Verbal IQ Index (DepVIQ), Gesell Development Schedule (GDS), Raven Colored Progressive Matrices Test (RCPM), Wechsler Intelligence Scale for Children IV (WISC-IV). One study had insufficient data to calculate 95% CI (32).

Children with a high pre-natal PAH exposure were found to have a delay in overall child intelligence [OR = 1.75, 95% CI, 1.11–2.71) (54), mental development [OR = 0.65 (32)], and average overall development (27, 44) (OR = 0.84, 95% CI, 0.52–1.36; OR = 1.85, 95% CI, 1.13–3.01, respectively). Specifically, the greatest negative effects reported were in verbal IQ (OR = 3.45, 95% CI, 0.95–12.49) (53) and language (OR = 5.99, 95% CI, 1.88–19.02) (47). However, the latter could not be confirmed in five out of six studies (27, 42, 44, 45, 56). Two studies analyzed the effect of PAH on general cognitive abilities with contradictory results: one (31) reported a negative effect (OR = 2.89, 95% CI, 1.33–6.25) while another (56) reported no effect. PAH effect on impaired motor development was inconclusive, as confirmed by four studies (27, 42, 44, 45) (OR = 0.95, 95% CI, 0.58–1.53; OR = 1.91, 95% CI, 1.22–2.97; OR = 1.63, 95% CI, 1.00–2.65; OR = 1.82, 95% CI, 3.21–1.03, respectively), whereas three others could not confirm it. No association was found between PAH exposure and developmental motor ability (56), fine and gross motor abilities (47), and psychomotor abilities (31). Only one study reported the effect of PAH and reduced adaptive development (27) (OR = 1.77, 95% CI, 1.09–2.88) while four out of five studies reported no association with adaptive domains (42, 44, 45, 47). Size effects reported by the studies mentioned are graphically represented in Figure 6 and listed in Table 1.

**Table 1:** Studies with measured pre-natal PAH exposure on cognitive abilities in childhood.

**References**	**Sample size**	**Sample characteristics**	**Male: female**	**Mean age (SD)**	**Age range**	**Comorbidities**	**Air pollution data acquisition method**
Perera et al. (31)	183	Children 3 years of age, mothers 18–35 years, non-smoking, free of diabetes, hypertension, or known HIV, African American and Dominican women residing for a minimum of a year in Washington Heights, Harlem, or the South Bronx in New York City	84:99	3.5 (0.5)	3 years to 3 years 12 months	N/A	Environmental samples analyzed for 8 PAHs
Jedrychowski et al. (53)	170	Children 7 years of age, mothers ≥18 years of age, non-smoking, singleton pregnancies, no history of illicit drug use, pregnancy related diabetes, or hypertension, no current occupational exposure to PAH or any other known developmental toxicants, and have been resident in Krakow, Poland for a minimum of a year	80:90	7.5 (0.5)	7 years to 7 years 12 months	N/A	Cord blood PAH–DNA adduct
Zhang et al. (47)	211	Infants 12 months of age, free from delivery injuries, neonatal problems, acquired disabilities, developmental dysplasia or other developmental defects, mothers resident in Qingdao city, China for at least 3 years, free from diabetes, known HIV and known neuropsychiatric disease.	192:156	1.0 (0.083)	1 year to 1 year 1 month	N/A	Cord blood benzo(a)pyrene-DNA adducts (ng/mL)
Perera et al. (32)	380	Children 2 years of age, mothers 18–35 years, non-smoking, free of diabetes, hypertension, or known HIV, African American and Dominican women residing for a minimum of a year in Washington Heights, Harlem, or the South Bronx in New York City.	N/A	2.5 (0.5)	2 years to 2 years 12 months	N/A	PAH/aromatic DNA adducts in umbilical cord blood samples
Polanska et al. (56)	406	Children 1–2 years of age, mothers had single pregnancy up to 12 weeks of gestation, no assisted conception, no pregnancy complications, no chronic disease, resident in Poland	192:214	1.5 (0.5)	1 year to 2 years 12 months	N/A	1-hydroxypyrene metabolites in mothers' urine
Edwards et al. (54)	214	Children 5 years of age, mothers ≥18 years of age, non-smoking, singleton pregnancies, no history of illicit drug use, pregnancy related diabetes, or hypertension, no current occupational exposure to PAH or any other known developmental toxicants, and have been resident in Krakow, Poland for a minimum of a year	103:111	5.5 (0.5)	5 years to 5 years 12 months	N/A	Environmental samples analyzed for 8 PAHs
Perera et al. (44)	217	Children 2 years of age, born between either March to June 2002 or March to May 2002, mothers ≥20 years, non-smoking, resident within 2 km of Tongliang power plant	113:104	2.5 (0.5)	2 years to 2 years 12 months	N/A	Cord blood benzo(a)pyrene-DNA adducts (ng/mL)
Tang et al. (45)	110	Children 2 years of age, born between March and June 2002, mothers ≥20 years, non-smoking, resident within 2 km of Tongliang power plant	54:56	2.5 (0.5)	2 years to 2 years 12 months	N/A	Cord blood benzo(a)pyrene-DNA adducts (ng/mL)
Tang et al. (26)	215	Children 2 years of age, born between either March to June 2002 or March to May 2002, mothers ≥20 years, non-smoking, resident within 2 km of Tongliang power plant	106:109	2.5 (0.5)	2 years to 2 years 12 months	N/A	Cord blood benzo(a)pyrene-DNA adducts (ng/mL)
Cao et al. (42)	158	Children 2 years of age, mothers ≥18 years of age, non-smoking, resident in Taiyuan, Shanxi province, China for a minimum of 1 year	82:76	2.5 (0.5)	2 years to 2 years 12 months	N/A	The sum of the maternal concentrations of eleven urinary PAHs metabolites Σ-OHPAHs

#### Association between pre-natal PAH exposure and neurobehavioral development

**Figure 7:**
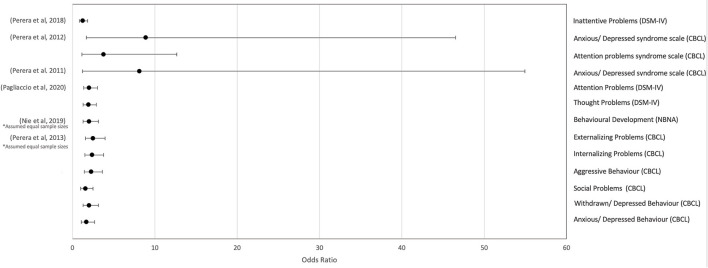
Forest plot of the calculated odds ratios (OR) and 95% confidence intervals (95% CI) for the association between pre-natal exposure to PAH and measures of neurobehavioral development. Gesell Development Schedule, Diagnostic (GDS) and Statistical Manual of Mental Disorders, 4th Edition (DSM-IV), Child Behavior Checklist (CBCL), Neonatal Behavioral Neurological Assessment (NBNA). *Sample sizes were assumed equal.

Children with a high pre-natal PAH exposure were found to exhibit externalizing and internalizing behavioral problems (OR = 2.49, 95% CI, 1.57–3.95; OR = 2.39, 95% CI, 1.51–3.79, respectively) (55), and infants exhibited a decrease in behavioral development (OR = 2, 95% CI, 1.27–3.15) (43). Associations with anxious/depressed behavior were found in three out of four studies (33, 34, 55) (OR = 8.89, 95% CI, 1.7–46.51; OR = 8.14, 95% CI, 1.21–54.94; OR = 1.7, 95% CI, 1.08–2.68, respectively), with no association found by one study (36). Three out of five studies reported a negative effect on children's attentiveness (33, 35, 36) (OR = 1.34, 95% CI, 0.85–1.83; OR = 2.02, 95%, 1.35–3.03; OR = 3.79, 95% CI, 1.14–12.66) whilst two (34, 55) reported no effect. The report from one study (55) about the effect of both withdrawn/depressed and aggressive behavior (OR = 2, 95% CI, 1.27–3.16; OR = 2.29, 95% CI, 1.45–3.62, respectively) was contradicted by another study (36) that reported no effect for either. The latter (36) did, however, report the effect of impaired thought problems (OR = 1.95, 95% CI, 1.3–2.91) which was contradicted by the former (55). Only one out of seven studies reported an association between PAH and social problems (55) (OR = 1.57, 95% CI, 1.00–2.48), and the remaining six reported no effect (27, 36, 42, 44, 45, 47). Two studies (36, 55) found no effect on rule breaking behavior or somatic complaints. One study (35) reported no associations with attention deficit hyperactivity disorder (ADHD) index scores or hyperactive compulsive behavior, nor did another from the same research group (31) regarding total behavioral problems. Studies reporting neurobehavioral effects are reported in Table 2, and effect sizes are depicted in Figure 7.

**Table 2:** Studies with measured pre-natal PAH exposure on neurobehavioral development.

**References**	**Sample size**	**Sample characteristics**	**Male: female**	**Mean age (SD)**	**Age range**	**Comorbidities**	**Air pollution data acquisition method**
Perera et al. (35)	351	Children 9 years of age, mothers 18–35 years, non-smoking, free of diabetes, hypertension, or known HIV, African American and Dominican women residing for a minimum of a year in Washington Heights, Harlem, or the South Bronx in New York City.	163:188	9.01 (0.19)	9 years to 9 years 12 months	N/A	Cord blood benzo(a)pyrene-DNA adducts (ng/mL)
Perera et al. (33)	253	Children 6–7 years of age, mothers 18–35 years, non-smoking, free of diabetes, hypertension, or known HIV, African American and Dominican women residing for a minimum of a year in Washington Heights, Harlem, or the South Bronx in New York City.	131:122	6.5 (0.5)	6 years to 7 years 12 months	N/A	Environmental samples analyzed for 8 PAHs
Perera et al. (34)	215	Children 3 years 9 months to 5 years 11 months of age, mothers 18–35 years, non-smoking, free of diabetes, hypertension, or known HIV, African American and Dominican women residing for a minimum of a year in Washington Heights, Harlem, or the South Bronx in New York City.	87:128	4.8 (not reported)	3 years 9 months to 5 years 11 months	N/A	Cord blood benzo(a)pyrene-DNA adducts (ng/mL)
Pagliaccio et al. (36)	319	Children 11 years old, mothers 18–35 years, non-smoking, free of diabetes, hypertension, or known HIV, African American and Dominican women residing for a minimum of a year in Washington Heights, Harlem, or the South Bronx in New York City.	177:142	11.5 (0.5)	11 years to 11 years 12 months	Early life stress	Environmental samples analyzed for 8 PAHs
Nie et al. (43)	247	Infants 3 days of age, mothers ≥18 years, non-smoking, no chronic disease or family history of neurological disease, single gestational viable fetus, who delivered in the Sixth Hospital of Shanxi Medical University and the Eighth People's Hospital of Taiyua, resident in Taiyuan for at least a year	132:115	3 days (not reported)	3 days	N/A	Urinary metabolite concentrations of 2-hydroxyfluorene
Perera et al. (55)	248	Children from Krakow, Poland, mothers ≥18 years, non-smoking	122:126	7.28 (0.98)	6 years to 9 years 12 months	Maternal psychological distress	Personal air monitoring analyzing concentrations of 8 PAHs
Tang et al. (45)	110	Children 2 years of age, born between March to June 2002, mothers ≥20 years, non-smoking, resident within 2 km of Tongliang power plant	54:56	2.5 (0.5)	2 years to 2 years 12 months	N/A	Cord blood benzo(a)pyrene-DNA adducts (ng/mL)
Zhang et al. (47)	211	Infants 12 months of age, free from delivery injuries, neonatal problems, acquired disabilities, developmental dysplasia or other developmental defects, mothers resident in Qingdao city, China for at least 3 years, free from diabetes, known HIV, and known neuropsychiatric disease.	192:156	1.0 (0.083)	1 year to 1 year 1 month	N/A	Cord blood benzo(a)pyrene-DNA adducts (ng/mL)
Perera et al. (44)	217	Children 2 years of age, born between either March to June 2002 or March to May 2002, mothers ≥20 years, non-smoking, resident within 2 km of Tongliang power plant	113:104	2.5 (0.5)	2 years to 2 years 12 months	N/A	Cord blood benzo(a)pyrene-DNA adducts (ng/mL)
Tang et al. (27)	215	Children 2 years of age, born between either: March to June 2002 or March to May 2002, mothers ≥20 years, non-smoking, resident within 2 km of Tongliang power plant	106:109	2.5 (0.5)	2 years to 2 years 12 months	N/A	Cord blood benzo(a)pyrene-DNA adducts (ng/mL)
Cao et al. (42)	158	Children 2 years of age, mothers ≥18 years of age, non-smoking, resident in Taiyuan, Shanxi province, China for a minimum of 1 year	82:76	2.5 (0.5)	2 years to 2 years 12 months	N/A	The sum of the maternal concentrations of 11 urinary PAHs metabolites Σ-OHPAHs
Perera et al. (31)	183	Children 3 years of age, mothers 18–35 years, non-smoking, free of diabetes, hypertension, or known HIV, African American and Dominican women residing for a minimum of a year in Washington Heights, Harlem, or the South Bronx in New York City	84:99	3.5 (0.5)	3 years to 3 years 12 months	N/A	Environmental samples analyzed for 8 PAHs

#### Association between pre-natal PM_2.5_ exposure and cognitive abilities and neurobehavioral development in childhood

A study (40) examined high PM_2.5_ exposure during early, mid, and late pregnancy with measures of full-scale IQ score, inattentiveness, and adverse memory performance. Boys highly exposed during late pregnancy exhibit lower IQ and inattentiveness when exposure was from mid to late pregnancy. Girls highly exposed during early to mid pregnancy exhibited adverse memory performance. No effect was reported for the remaining domains analyzed by this study (40).

The finding of impaired motor development (48) was not supported by a subsequent study conducted by the same group (49), which reported, however, impaired memory in boys (49). From studies analyzing the impact of pre-natal PM_2.5_ exposure on cognition and neurobehavioral development (Table 3), no effect was found on visual-motor functioning (61), general cognitive ability (28, 49), mental status (48), non-verbal intelligence (61), adaptive function or autism spectrum disorder (28), nor on verbal, perceptive manipulative, and numeric development (49).

**Table 3:** Studies with measured pre-natal PM_2.5_ exposure on cognitive abilities and neurobehavioral development in childhood.

**References**	**Sample size**	**Sample characteristics**	**Male: female**	**Mean age (SD)**	**Age range**	**Comorbidities**	**Air pollution data acquisition method**
Blazkova et al. (61)	169	Children 5 years of age, born in the summer 2013 to winter 2014, non-smoking mothers, resident in Karvina and Ceske Budejovice, Czech Republic	78:90	5.5 (0.5)	5 years to 5 years 12 months	Viral diseases otitis bronchitis GIS HCD	Analysis of 11 OH-PAHs in urine
Kerin et al. (28)	325	Children 2–5 years, resident in catchment area of 20 counties in northern California, the central valley and parts of Los Angeles metropolitan area, US, complete history of environmental air exposure, lived with at least 1 biological parent who speaks English or Spanish.	281:44	(not reported)	2 years to 5 years 12 months	N/A	Residential addresses inputted into Tele Atlas database and software
Lertxundi et al. (49)	560	Children 4 years male, mothers ≥16 years, resident in Valencia, Sabadell, and Gipuzkoa in Spain	560:00	4.8 (4.9)	4 years to 4 years 12 months	N/A	Land use regression models
Lertxundi et al. (48)	438	Children aged ~15 months age, mothers ≥16 years, singleton pregnancies	198:240	1.25 (0.25)	1 year 1 month to 1 year 6 months	N/A	Environmental samples from digital DHA-80 high-volume aerosol samplers
Chiu et al. (40)	119	Mothers ≥18 years, at 28.4 ± 7.9 weeks gestation between August 2002 and January 2000 in Boston.	00:119	6.5 (0.98)	6 years to 7 years 3 months	N/A	Use of a hybrid satellite based spatio-temporal prediction model and residential address during pregnancy

### Childhood

#### Association between childhood PAH exposure and cognitive abilities and neurobehavioral development

Children exposed to high levels of PAH post-natally exhibited lower general cognitive ability and delayed impaired memory (62). Increased inattentiveness was reported by two studies (50, 62), but this finding was contradicted by one study (51). The negative effect of post-natal PAH exposure was not observed in all memory domains. A study (50) found an association between impaired working numeric memory but not on working verbal memory. Short-term memory was not found affected either (62).

The association with ADHD diagnosis reported by one study (38) was not supported by two other studies (50, 51). Neither study found an effect on learning performance (38, 62) or an association with visual spatial skills, non-verbal test performance, executive function, motor performance (62), or behavioral problems (50). Studies reporting childhood PAH exposure can be found in Table 4.

**Table 4:** Studies with measured childhood PAH exposure on cognitive abilities and neurobehavioral development.

**References**	**Sample size**	**Sample characteristics**	**Male: female**	**Mean age (SD)**	**Age range**	**Comorbidities**	**Air pollution data acquisition method**
Suter et al. (62)	31	Children aged 5–12 resident in Nairobi, Kenya, Infected with HIV and previously enrolled in the Optimizing HIV-1 Therapy Study	N/A	6.6 (0.8)	5 years to 12 years 12 months	HIV	Concentration of urinary PAH metabolite 1-hydroxypyrene (1-OHP)
Mortamais et al. (51)	242	Children 7–10 years, resident and enrolled in one of 40 schools in Barcelona, Spain, no dental braces	123:119	8.4 (0.8)	7 years to 10 years 12 months	N/A	Environmental air sampling
Abid et al. (38)	83	Children 6–15 years of age, part of a civilian population resident in the US	58:25	11.2 (0.5)	6 years to 15 years 12 months	N/A	Urinary metabolite concentrations of 2-napthol
Alemany et al. (50)	1589	Children aged 7–11, attending one of 38 schools in Barcelona, Spain, and one school in the adjacent municipality, Sant Cugat del Vallés	831: 758	8.52 (0.87)	7 years to 11 years 12 months	APOE e4 allele	Environmental samples analyzed for 7 PAHs

#### Association between childhood PM_2.5_ exposure and cognitive abilities and neurobehavioral development

From the four studies reporting post-natal PM_2.5_ exposure on general cognition and neurobehavior (Table 5), one reported that children exposed to high levels of PM_2.5_ post-natally displayed impaired selective and sustained attention (63), but this finding was contradicted by two studies (39, 52) reporting no effect on inattentiveness or attention and executive function, respectively. A study's report of impaired visual information processing speed (63) was again contradicted by another (39) reporting no association with processing speed. No association was found with working memory (39, 52), episodic memory, language (39), cognitive ability, adaptive function, or autism spectrum disorder (28).

**Table 5:** Studies with measured childhood PM_2.5_ exposure on cognitive abilities and neurobehavioral development.

**References**	**Sample size**	**Sample characteristics**	**Male: female**	**Mean age (SD)**	**Age range**	**Comorbidities**	**Air pollution data acquisition method**
Cserbik et al. (39)	10, 343	Children aged 9–10 years, resident in one of 21 study sites in the US	5,410: 4,933	9.93 (0.64)	9 years to 10 years and 12 months	N/A	Ensemble-based model approach combining aerosol optical depth models, land-use regression, and chemical transport models
Kerin et al. (28)	325	Children 2–5 years, resident in catchment area of 20 counties in northern California, the central valley and parts of Los Angeles metropolitan area, US, complete history of environmental air exposure, lived with at least one biological parent who speaks English or Spanish	281:44	(Not reported)	2 years to 5 years 12 months	N/A	Residential addresses inputted into Tele Atlas database and software
Rivas et al. (52)	2,221	Children 7–10 years old, attending one of 39 schools in Barcelona, Catalonia, Spain, without special needs	1,133: 1,088	8.5 (0.9)	7 years to 10 years 12 months	N/A	Land use regression models
Saenen et al. (63)	310	Children in grades 3–6 in three primary schools, Flanders, Belgium.	158: 152	10.2 (1.3)	N/A	N/A	Chronic exposure: spatial temporal interpolation method to model the daily residential exposure. Recent exposure (at schools): portable devices

#### Association between childhood PM_2.5_ exposure and neurodegeneration

Only three studies by the same research group analyzed post-natal PM_2.5_ exposure to neurodegeneration (Table 6). Two of them found that children highly exposed to PM_2.5_ post-natally exhibited lower amyloid beta protein fragment 1–42 (Aβ_1 − 42_) and brain-derived neurotrophic factor (BDNF) (29, 30), with one finding, in addition, higher interferon (IFN) γ concentrations in cerebrospinal fluid (CSF) (30). No effect was found with regard to concentrations of biomarkers: non-phosphorylated tau (non-p-tau), vitamin D, tau phosphorylated at threonine 181 (30), cellular prion protein, total tau, interleukin (IL) β, leptin (29, 30), total alpha- synuclein (α-synuclein), oligodendrocyte α-synuclein, hyperphosphorylated tau, tumor necrosis factor alpha, IL 2, IL 6, IL 10, or monocyte chemoattractant protein-1 (MCP-1) (29). Of 33 antibodies to neural and tight junction proteins, actin immunoglobulin G (IgG), occludin/zonulin (OZ) immunoglobulin A (IgA), OZ IgG, myelin oligodendrocyte glycoprotein (MOG) IgG, MOG immunoglobulin M (IgM), myelin basic protein (MBP) IgA, MBP IgG, astrocytic protein (S-100) IgG, S-100 IgM and cerebellar antigen (CEREB) IgG in serum, and MBP antibodies in CSF were higher in children exposed to high levels of PAH compared to controls (58).

**Table 6:** Studies with measured childhood PM_2.5_ exposure on neurodegeneration.

**References**	**Sample size**	**Sample characteristics**	**Male: female**	**Mean age (SD)**	**Age range**	**Comorbidities**	**Air pollution data acquisition method**
Calderón-Garcidueñas et al. (30)	1) 426 2) 81	Children admitted to Mexico City hospital, resident in Mexico City Metropolitan area (MCMA) and other small cities in Mexico	1) 256:161 2) 44:33	1) 13.36 (8.82) 2) 11.54 (5.1)	(Not reported)	Lymphoblastic leukemia	Environmental air sampling, for regulating levels above the USEPA standards
Calderón-Garcidueñas et al. (22)	1) 73 2) 126	Children admitted to Mexico City hospital, resident in Mexico City Metropolitan area (MCMA) and other small cities in Mexico	1) 42:31 2) 59:70	1) 11.7 (5.14) 2) 17.49 (15.98)	(Not reported)	Lymphoblastic leukemia	Environmental air sampling
Calderón-Garcidueñas et al. (58)	111	Children within 5 miles of Mexico City Metropolitan Area (MCMA) or small control cities in Mexico (Zacatlán and Huachinango, Puebla; Zitaácuaro, Michoacaán; Puerto Escondido, Oaxaca; Chalma, Veracruz; Tlaxcala, Tlaxcala), No ETS exposure, lived within 5 miles of an air monitoring station	54:57	13.37 (4.2)	(Not reported)	N/A	Environmental air sampling

### Adult

#### Association between adult PAH exposure and cognitive abilities

Adults highly exposed to PAH exhibited impaired auditory memory (41, 46), individual accounts of memory disturbances (60), and impaired verbal learning and memory (59). However, there was no effect on working memory and executive function, visuospatial memory/attention and planning (59), or visual perception memory (41, 46).

There was one account of impaired cognitive disturbances (60), which was contradicted by two reports of no association with cognitive dysfunction (37, 59) and by additional individual accounts of no effect in approximate number system functioning (41) (i.e., digit span, digit symbol, number of dots tests), confrontational word retrieval, verbal fluency, delayed reaction time between congruent and incongruent stimuli, visual attention, and task switching (59). Mood state, attention/response speed, manual dexterity, or perceptual motor speed were not found associated with PAH exposure in adulthood (41, 46). However, it must be noted that two investigations were population studies (37, 59), while the other three (41, 46, 60) were occupational health studies on brain effects of PAHs in coke ovens (41, 46) or oil spill (60) workers, who are exposed to very high levels of PAHs, especially high molecular weight compounds including benzo(a)pyrene and other compounds with five to six or more hydrocarbon rings. A summary of the population characteristics of the five studies exploring adult PAH exposure and general cognition can be found in Table 7.

**Table 7:** Studies with measured adult PAH exposure on cognitive abilities.

**References**	**Sample size**	**Sample characteristics**	**Male: female**	**Mean age (SD)**	**Age range**	**Comorbidities**	**Air pollution data acquisition method**
Du et al. (46)	697	Employed at a coking plant in Shanxi province, China for minimum of 1 year	470:227	39.73 (9.74)	24–64 years 12 months	N/A	The sum of the concentrations of 11 urinary PAHs metabolites Σ-OHPAHs
Cho et al. (59)	949	≥50 year-old individuals, no known neurological diseases, resident in Seoul, Incheon, Wonju, and Pyeongchang, Republic of Korea.	421:528	67.24 (6.39)	≥50 years	Hypertension diabetes dyslipidemia angina myocardial infarction	Concentrations of urinary PAHs metabolites including: 1-hydroxypyrene
Ha et al. (60)	565	Volunteers in the Hebei Spirit oil spill, 2007, near the shore of Taean, Korea.	275:288	N/A	N/A	Asthma	1-hydroxypyrene and 2-naphthol metabolites in urine
Niu et al. (41)	176	Male 23–48-year-old coke oven workers Taiyuan, China, employed for a minimum of 1 year, middle school educated.	176:00	37.86 (6.61)	23 years to 48 years 12 months	N/A	Concentration of urinary PAH metabolite 1-hydroxypyrene (1-OHP)
Best et al. (37)	454	≥60-year-old individuals without known neurological diseases, resident in 15 randomly selected states in the US	221:233	70.1 (0.02)	≥ 60 years	Hypertension thyroid disease stroke kidney disease liver disease	The sum of the concentrations of eight urinary PAHs metabolites (Σ-OHPAHs)

### Childhood and adult

#### Association between childhood and adult PM_2.5_ exposure and neurodegeneration

Details from the three studies reporting on cohorts inclusive of participants exposed to PM_2.5_ only during childhood and some participants through to adulthood can be found in Table 8. In a cohort of mixed exposure to PM_2.5_, the presence of neurodegenerative biomarkers phosphorylated tau (p-tau), α-synuclein, and transactive response DNA-binding protein 43 (TDP-43) was confirmed in brainstems (57). The faster increase in concentrations with regard to the age of non-p-tau in CSF was also associated with increased exposure (30). However, no association was found with regard to the concentration of total and oligomer α-synuclein in CSF (29).

**Table 8:** Studies with measured cohorts inclusive of childhood and adult PM_2.5_ exposure on neurodegeneration.

**References**	**Sample size**	**Sample characteristics**	**Male: female**	**Mean age (SD)**	**Age range**	**Comorbidities**	**Air pollution data acquisition method**
Calderón-Garcidueñas et al. (57)	186	Metropolitan Mexico City residents, acute cause of death not involving the brain, autopsies were performed 3.7 ± 1.7 h after death, autopsy material examined between 2004 and 2008	162:186	27.29 (11.8)	11 months to 41 years	N/A	Ministry of environment of Mexico city monitoring stations
Calderón-Garcidueñas et al. (30)	1) 426 2) 81	Children admitted to Mexico City hospital, resident in Mexico City Metropolitan area (MCMA) and other small cities in Mexico	1) 256:161 2) 44:33	1) 13.36 (8.82) 2) 11.54 (5.1)	N/A	Lymphoblastic leukemia	Environmental air sampling, for regulating levels above the USEPA standards
Calderón-Garcidueñas et al. (22)	1) 73 2) 126	Children admitted to Mexico City hospital, resident in Mexico City Metropolitan area (MCMA) and other small cities in Mexico	1) 42:31 2) 59:70	1) 11.7 (5.14) 2) 17.49 (15.98)	N/A	Lymphoblastic leukemia	Environmental air sampling

#### Risk of bias within studies

All studies included were of high quality with reproducible accounts of the method employed to assess relevant outcomes, and the inclusion/exclusion criteria used to select the study population were explained in sufficient detail (refer to detailed QUADAS tool responses in the publicly available data at https://datashare.ed.ac.uk/handle/10283/3892). Where applicable, all studies provided explanations for participant withdrawal, which were unrelated to both the exposure and the outcome being measured and reported intermediate or unexpected results. Approximately 54% of studies involved the use of a comparison with a low exposure or control population either by dichotomizing exposure data or using a demographically matched control population. The remaining 46% of studies assessed PAH exposure as a continuous variable. All studies correctly identified confounding variables, and the method and analysis were adjusted accordingly. There was, however, a considerable risk of information bias amongst studies, with only 16% of studies reporting the outcome assessor to be blinded and unaware of the exposure status of the study participant. Seventy-three percent of the studies provided no indication as to whether they were or not blinded, and in 11% the outcome assessors were confirmed not blinded.

## Discussion

This review found sufficient evidence that pre-natal PAH exposure negatively impacts cognitive function with specific regard to child intelligence, mental development, verbal IQ, memory impairment, average overall development, child attentiveness, behavioral development, and externalizing, internalizing, anxious, and depressed behavioral problems.

Evidence concerning exposure during childhood and as an adult with cognitive function was insufficient to conduct a meta-analysis, due to a reduced number of studies, low consistency, and high heterogeneity in results. However, associations can be observed such as exposure during childhood with lowered cognitive ability, impaired child attentiveness, and exposure as an adult manifesting in memory disturbances with specific regard to auditory memory and verbal learning and memory.

Studies concerning PAH exposure during childhood and as an adult were scarce, but an increased risk of neurodegeneration was found through the presence of neurodegenerative biomarkers and increased concentrations of cryptic “self” antigens in serum and CSF, indicative of the neuroinflammatory pathology which precedes Alzheimer's disease (AD) and Parkinson's disease (PD).

It is known that some pathways of aryl-hydrocarbon neurotoxicity are common for PAHs, TCDD, dioxin-like agents, polyphenols, and similar xenobiotics. A review of the neuropathological mechanisms of PAHs highlights that these, together with their metabolites, may cross the blood–brain barrier causing neurological abnormalities that may include neuronal damage, impaired neurotransmitter regulation, parasympathetic dysregulation, and neurodegeneration (65). Preclinical studies hint at a common neuropathological mechanism of PAH action being the binding of these compounds to the aryl-hydrocarbon receptor (AhR), a cytosolic transcription factor that initiates a complex pathway leading to alteration of gene regulation. AhR is also present in neural cells and can be involved in the mechanisms leading to PAH-induced neurological disorders (65).

This review differentially addressed the neurological impact of PAHs in three different domains, namely, cognitive abilities, neurobehavioral development, and neurodegeneration, and can be used as evidence for policy surrounding the monitoring of PAHs specifically. In addition, it raises awareness of the potentially confounding effect that different ambient PAH concentrations, in metropolitan and rural settings, can have on research assessing outcomes concerned with cognitive function and neurodegeneration in studies. It was not possible, however, to conclude on the differential impact of PAHs acquired mainly from outdoor sources from those acquired from indoor sources.

A previous review on the impact of PM_2.5_ in disease incidence did not stratify patients by age nor considered differences between urban and rural areas, rather stratifying studies by the pollution level in which the country was considered (i.e., “lightly polluted” vs. “heavily polluted”) (64). Other reviews have highlighted general adverse health conditions such as chronic asthma, increased incidence of premature death and hospital admissions (15), and kidney and liver damage (16). Some focused specifically on the carcinogenic properties and resulting incidence of the lung (17), urinary tract (18), and skin and gastrointestinal tract cancers (16). Those that focused on the neurological impact of air pollution concerned a diverse mixture of compounds. One specifically focused on non-communicable diseases and the roles of nitrogen dioxide (NO_2_), nitrogen oxide species (NO_x_), carbon monoxide (CO), and PM_2.5_ (19). Another (20) raises awareness concerning ambient pollution's adverse effect on cognitive decline and impairment, concurring with findings from (22), where the emphasis was on ozone, PM_2.5_, and PM_10_. A study (21) reported NO_2_, NO_x_, black carbon, and PMs as potential risk factors for AD, PD, and multiple sclerosis. Despite the outcomes assessed being orientated toward neurological health, the exposures measured either include multiple pollutants or compounds NO_2_, NO_x_, CO, PM, or black carbon, around which extensive research already exists and has culminated in tight air quality restrictions and monitoring, which is closely adhered to by governing bodies. This review raises awareness of the neurological impact PAH has, independent of other pollutants, the importance of which is paramount with the current health impacts of PAHs in the UK air quality strategy detailed as “possibly” or “probably” carcinogenic, detracting from the seriousness of their impact on neurological human health (2). This review proceeds to categorize outcomes into subgroups depending on the time of exposure to provide further insight into the demographics of the individuals most vulnerable to the pollution levels reported and to differentiate between the areas of cognitive function and neurodegeneration most impacted, elucidating the potential mechanisms of neurotoxicity. The observation that the most profound effect of PAH exposure culminates from the pre-natal period is in keeping with prior research, showing the fetal brain to be more vulnerable to environmental toxic insult than the adult. The increased permeability of the not yet fully formed blood–brain barrier combined with the rapid brain growth during the second trimester means the period of most intense construction and brain architecture is also the time the brain is most vulnerable to the passage of toxins and neurotoxicity (66). Overall, this review has systematically located, summarized, and meta-analyzed evidence about the potent neurotoxicity of direct or indirect exposure to PAHs across the human lifespan, highlighting the need for more well-designed epidemiological studies.

### Limitations

Studies included in the analysis were limited to those written in the English language. Publication bias and selective reporting within studies cannot be discarded, nor can indexing issues, in which the search terms may have failed to identify relevant studies.

The study populations included only originated from nine countries, of which the UK was not one. Findings are therefore limited to the environments and seasonal variations in climate found in these countries, and no specific recommendations for the UK, where the present review was conducted, can be made. The studies included also involved the use of different subgroup samples from the same large cohort, due to the necessity and availability of a limited number of longitudinal study databases. Sampling bias cannot, therefore, be disregarded.

Other polluting aryl-hydrocarbons present in the air, in particulate matter (PM_2.5_) and diets, such as 2,3,7,8-tetrachlorodibenzo-p-dioxin (TCDD) and its congeners, dibenzofurans and dioxin-like polychlorinated biphenyls, have been reported to induce similar neurotoxicity and neurological disorders to PAHs. The concomitant exposure to these compounds, which are ubiquitously present as persistent organic pollutants, could have confounded the measured effects of PAHs reported in the studies reviewed. If the primary sources did not disentangle their effects, it is possible that some of the meta-analyzed results embody added effects of these aryl-hydrocarbons on ambient PAHs.

To adjust for heterogeneity, studies were stratified depending on the time point of exposure and outcome assessed; however, this did not account for heterogeneity between evaluators and instruments used, mainly due to the limited number of sources analyzed. In addition to this, the use of five different measures to quantify levels of PAH exposure, as well as the inclusion of quantification of PM_2.5_ as a measure, resulted in heterogeneity in exposure measurement instruments and the inclusion of potential contaminating compounds within PM_2.5_, which would confound results. Finally, there were insufficient data to calculate 95% CI for one study (32), and the request for numeric data from another study received no response (60); hence, the report of an effect on memory and cognitive disturbances was inferred from a figure with no confirmation from the raw data.

### Future research

The role of biological sex in the neurotoxic effects of PAH exposure requires further investigation. Sex stratification of data concerning memory impairment in pre-natally exposed populations was contradictory. Further accounts of memory impairment following both childhood and adult exposure should be dichotomized to examine sensitivity between sexes. Pre-natal PAH exposure's effect on motor development was an area of controversy. Additional research is required in this domain to eliminate ambiguity. Individual reports of a lack of association with motor performance and perceptual motor speed, respectively, were inadequate to clarify such controversy or draw any conclusions.

In addition to this, a more thorough examination of the timescale of PAH exposure is needed, utilizing a smaller scale to determine critical windows.

Stratification by pregnancy term elucidated differential full-scale IQ, inattentiveness, and memory performance results. No effect on the concentration of non-p-tau in CSF was reported following childhood exposure, however when looked at in a mixed cohort of childhood and adult exposure, as association in relation to age progression was reported, indicative of a critical window of exposure.

Furthermore, gene-environment interactions need further analysis, for example PAHs effect on the brain of genetically susceptible populations, such as carriers of the APOE4 allele.

Repeated future analysis on longitudinal cohorts is required to examine the impact of sustained high PAH exposure or subsequent markedly reduced exposure, the effects such fluctuations can have on cognitive function and neurodegeneration, and whether some adverse effects from pre-natal or early life exposure are recoverable.

Future research should identify and analyze the individual contributions and specific synergistic combinations of PAHs on neurological health. This would differentiate and determine the most neurotoxic PAHs and provide evidence for updates in policy, requiring the monitoring of additional PAHs, rather than only B(a)P. Additional research into the threshold at which PAH is capable of exerting neurotoxic effects would inform policy, with scientific backing to implement a safe limit with regard to neurological health and update the limit of 0.25 ng.m^−3^ B(a)P, which was set only with regard to carcinogenic properties. Furthermore, more studies are needed concerning populations in the UK, to account for the local environmental, climate, and seasonal variations capable of altering PAH's neurotoxic properties.

## Data availability statement

The datasets presented in this study can be found in online repositories. The name of the repository and accession number can be found below: Edinburgh DataShare, https://datashare.ed.ac.uk/, https://doi.org/10.7488/ds/3031.

## Author contributions

All authors listed have made a substantial, direct, and intellectual contribution to the work and approved it for publication.

## Footnotes

^1^QUADAS-2. URL http://www.bristol.ac.uk/population-health-sciences/projects/quadas/quadas-2/.

## Supplementary material

Supplementary Figure 1 Anthropogenic sources (industrial, mobile, domestic and agricultural sources) of PAH release into the environment (figure made using: Biorender.com).
