# Supplementary figures and images for: Impact of polycyclic aromatic hydrocarbon exposure on cognitive function and neurodegeneration in humans: A systematic review and meta-analysis
Source: Front Neurol. 2023 Jan 10;13:1052333. doi: 10.3389/fneur.2022.1052333 (PMC9871581; doi:10.3389/fneur.2022.1052333)

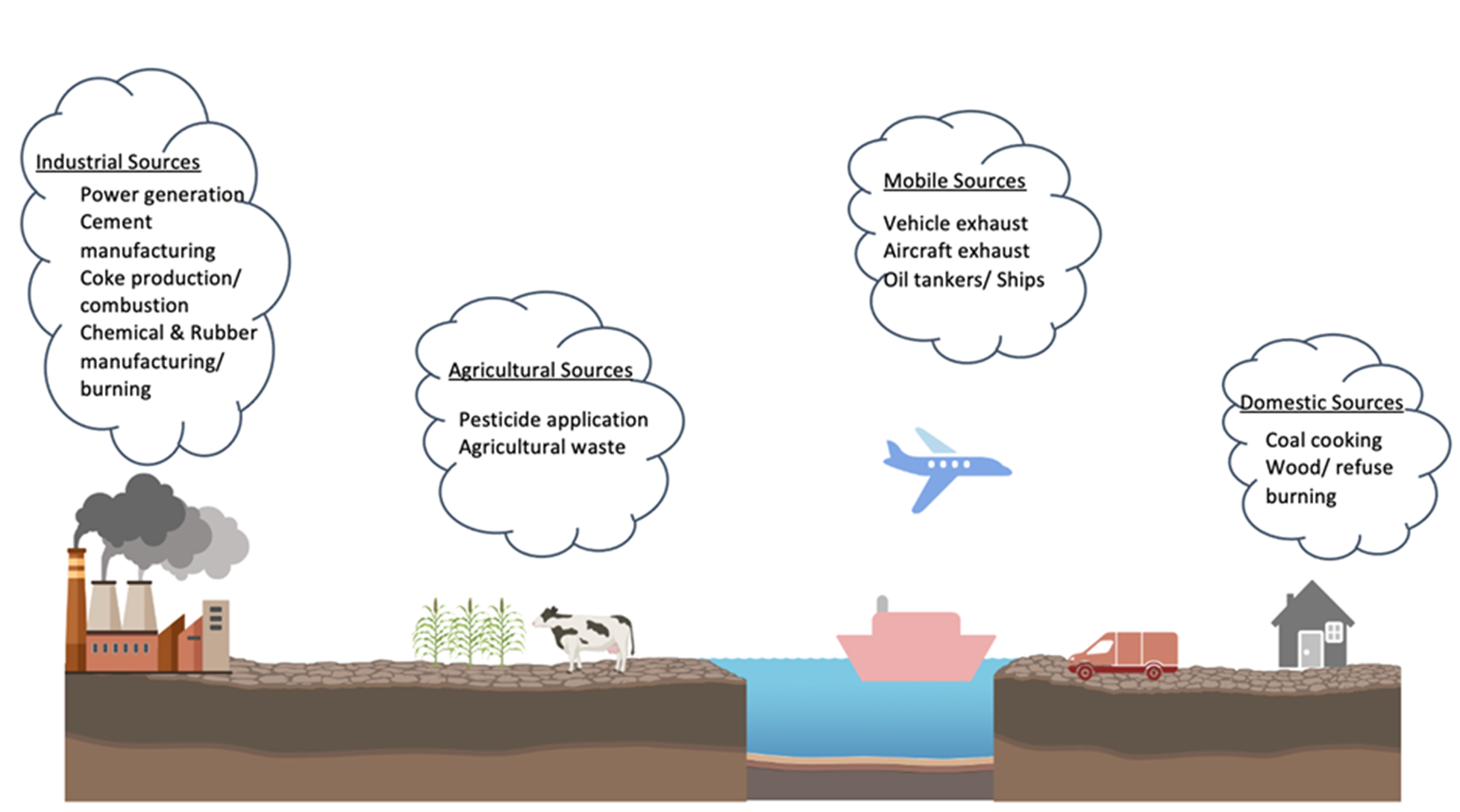

Supplement: Supplementary Figure 1 — Anthropogenic sources (industrial, mobile, domestic and agricultural sources) of PAH release into the environment (figure made using: Biorender.com). [file Image_1.TIF]
